# Utilization and implementation of remote monitoring of cardiac implantable electronic devices in Australia and New Zealand: Adoption, workload, and integration challenges

**DOI:** 10.1016/j.hroo.2025.12.004

**Published:** 2025-12-13

**Authors:** Brodie Sheahen, Edel O’hagan, Kenneth Cho, Kam Cheung Wong, Pierre Qian, Aravinda Thiagalingam, Graham Hillis, James Marangou, Derek Chew, Simone Marschner, Haeri Min, Liliana Laranjo, Clara Chow

**Affiliations:** 1 Westmead Applied Research Centre, The University of Sydney, Westmead Hospital, Westmead, NSW, Australia; 2 Westmead Hospital, Cardiology Department, Westmead, NSW, Australia; 3 Royal Perth Hospital, Cardiology Department, Perth, WA, Australia; 4 Cardiology Department, Victorian Heart Hospital, Clayton, VIC, Australia

**Keywords:** Cardiac implantable electronic devices, Remote monitoring, Health care services, Care models, Implantation rates

## Abstract

**Background:**

Remote monitoring (RM) of cardiac implantable electronic devices (CIEDs) reduces health care visits and improves clinical outcomes, but its use and service models are poorly characterized.

**Objective:**

This study aimed to examine the current landscape of CIED RM in Australia and New Zealand, including the prevalence of CIED and RM use, care models for RM data management, and infrastructure and resource requirements.

**Methods:**

A cross-sectional study was conducted using surveys of CIED clinics in Australia and New Zealand and analysis of deidentified CIED industry data. Data were obtained from public and private clinics and all 5 major CIED manufacturers.

**Results:**

Operational data were obtained from 50 clinics and implant/RM distribution data from all 5 manufacturers. From 2019 to 2023, total CIED implantations increased by 10.2% (125.9–138.8 per 100,000 people), whereas RM transmitter distribution rose 55.8% (75.5–117.6 per 100,000 people). In 2023, RM use was highest in remote (88.4%) and rural regions (85.3%) compared with regional (73.1%) and metropolitan (80.3%) areas. From survey data, the median clinic patient load was 1303 patients (interquartile range 1820) per year. On average, 30.2% of clinic workload (standard deviation 21.8%) was dedicated to managing RM alerts, primarily by cardiac physiologists (90.5%). The mean interval between scheduled in-person CIED follow-ups was longer for RM patients than non-RM patients (10.0 vs 7.2 months; *P* < .001).

**Conclusion:**

This study provides the first comprehensive analysis of CIED RM service use, offering insights to inform future RM care models and the development of other virtual care systems using remote patient data.

**Key Findings**

▪ From 2019 to 2023, cardiac implantable electronic device (CIED) implants in Australia increased by 16.5%, rising from 32,145 to 37,437 procedures.
▪ During the same period, the percentage of CIEDs issued with remote monitoring (RM) transmitters increased from 59.9% (19,259 of 32,145) in 2019 to 84.7% (31,711 of 37,437) in 2023.
▪ RM use in the public sector increased from 25.9% to 65.0%; however, the use rate remained lower compared with the private sector, which increased from 83.1% to 91.6%.
▪ On average, CIED clinics dedicate 30.2% of their workload to managing RM.
▪ CIED patients with RM had longer intervals between in-person follow-ups than those without RM (10.0 vs 7.2 months; *P* < .001).

## Introduction

Cardiac implantable electronic device (CIED) use is increasing in Australia, driven by expanding clinical indications and the aging population.^1^ Remote monitoring (RM) technology enables transmission of CIED data to health care teams in remote locations. Introduced in 2001, RM has demonstrated benefits, including fewer hospitalizations, earlier detection and management of clinical issues, reduced atrial fibrillation–related strokes, and improved heart failure care.^2^, ^3^, ^4^ RM availability has reshaped health services for CIED patients; however, there is little research on real-world RM utilization, service implementation, and variation across settings.^5^ In 2024, estimates from Australian Medical Benefits Scheme data reported that 19% of private-sector permanent pacemaker (PPM) patients used RM. Still, this figure is unlikely to reflect all RM use, given that Medical Benefits Scheme items for RM are capped and only applicable to certain types of RM use as a replacement for a CIED follow-up visit.^5^ Internationally, and in Australia, models of care, infrastructure, staffing, and funding for RM vary widely.^2^^,^^3^^,^^6^ Understanding RM services is critical for planning future health systems and the workforce to support CIED management.

The RM landscape for CIEDs in Australia and New Zealand remains poorly defined. This study aimed to characterize current and rapidly evolving CIED RM use in Australia and New Zealand, with implications for models of care and appropriate allocation of infrastructure and resources.

## Methods

### Study overview

This was a cross-sectional analysis of CIED and RM distribution data from all 5 CIED manufacturers supplying CIEDs in Australia and operational data from CIED clinics throughout Australia and New Zealand. This study is a component of the REMOTE-CARE program, which includes 5 studies with the overall aim to improve outcomes for patients with CIED on RM in Australia (Medical Research Future Fund grant number: 2025201). The program is supported by stakeholders such as the national cardiology body, Cardiac Society of Australia and New Zealand (CSANZ); the Medical Technology Association of Australia (MTAA) cardiac forum, which is the national association representing the 5 large cardiac device companies (Abbott, Biotronik, Boston Scientific, Medtronic, and MicroPort); and Professionals in Cardiac Sciences Australia (PiCSA), the national representative body for cardiac physiologists. A cardiac physiologist delivers clinical, technical, and scientific services that assist in diagnosing and treating heart disease. The role replaces the former titles of cardiac technician and cardiac scientist and is endorsed by Australia’s peak representative body for the profession, PiCSA.^7^

### Data sources

**Figure 1:**
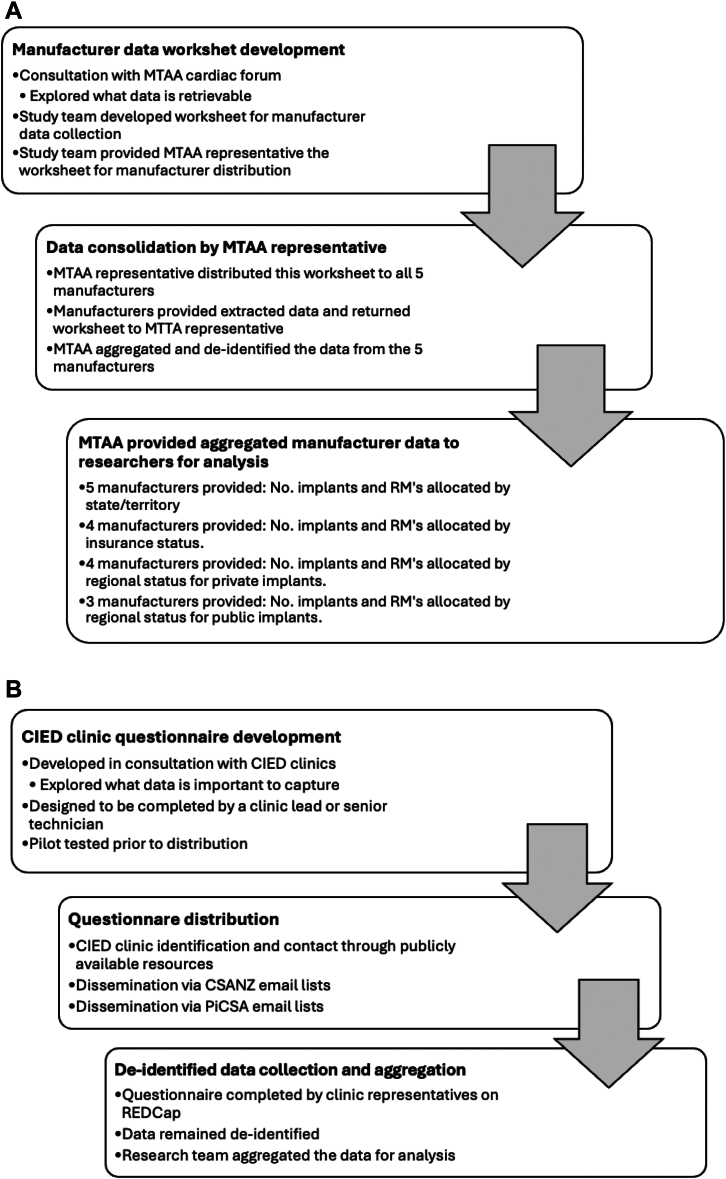
**A:** Process in collecting CIED manufacturer data. **B:** Process in collecting CIED clinic data. CIED = cardiac implantable electronic device; MTAA = Medical Technology Association of Australia; RM = remote monitoring.

2 data collection tools were used. 1 tool was provided to industry representatives to obtain deidentified industry data on CIEDs and RMs via the MTAA cardiac forum, which represents all manufacturer-supplied CIEDs in Australia (Figure 1A). MTAA liaised with the industry groups, and through this consultation, it was decided that, to balance feasibility and capture change, data would be extracted at 2 time points corresponding to the calendar years 2019 and 2023, representing the periods before and after the coronavirus disease 2019 pandemic, respectively. Data points included the number of CIED implants, the number of RMs allocated, the geographic location (remote/rural/regional/metropolitan) as determined by the modified Monash model^8^ and Australian state or territory (subnational jurisdictions), and insurance status (private or public) associated with the implant (Supplemental Appendix 1). The MTAA cardiac forum representative aggregated the data using a precoded worksheet that extracted and combined fields from the separate industry data sheets. The aggregated data were passed to the research team. This process maintained manufacturer privacy and enabled independent analysis by the research team. Data variables from CIED manufacturers were not uniform because industries differed in their internal data collection processes (Figure 1A).

The second tool was provided to CIED clinic representatives to obtain operational-level data on CIED RM (Figure 1B). CIED clinic data were obtained using a standardized questionnaire (Supplemental Appendix 2). Data points included self-reported time dedicated to supporting RM, clinic staffing, the clinical escalation process, and business practices. CIED clinics were identified from CIED manufacturers, Internet searches, CSANZ, and PiCSA through the dissemination of study details to their members. This survey was distributed to all clinic locations identified through the CSANZ and PiCSA e-mail lists, across all states and territories in Australia and New Zealand.

### Statistical analysis

Analyses were conducted using R statistical software (V4.2.0). Continuous variables are presented as mean and standard deviation based on the Shapiro-Wilk test of normality. Categorical variables are presented as frequencies and percentages. To determine the increase in CIED utilization between 2019 and 2023 relative to population growth, implantation data were calculated as a proportion of the Australian population on December 31, 2019 (25,522,169), and December 31, 2023 (26,966,789).

### Ethics

This study adhered to the principles of the Declaration of Helsinki. Given that only deidentified industry data and organizational-level survey responses were collected, the University of Sydney ethics committee determined that this study was exempt under national human research guidelines (Supplemental Appendix 3).

## Results

### CIED industry data

**Figure 2:**
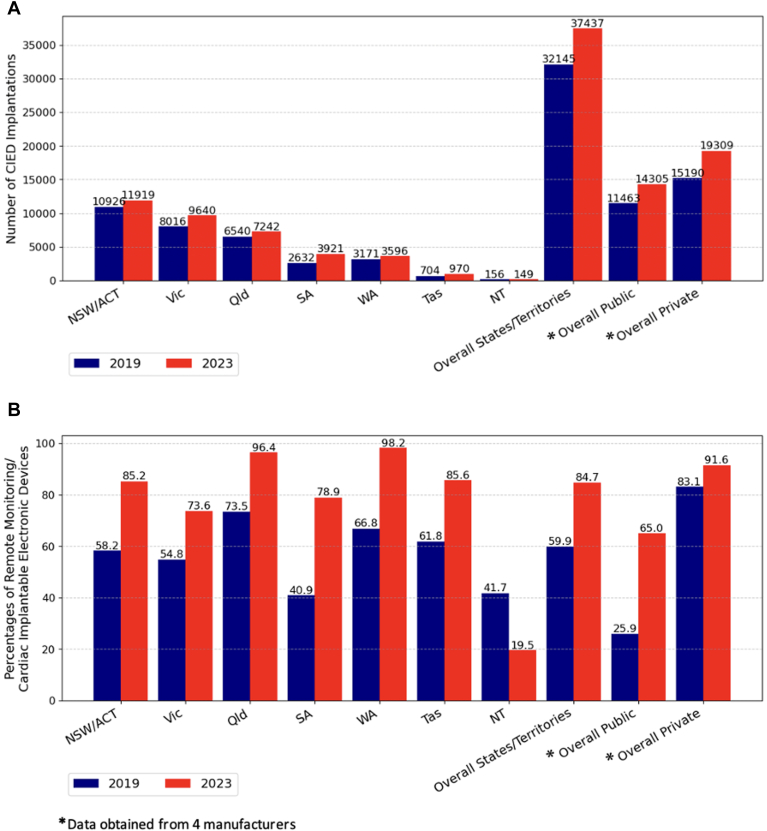
**A:** Number of CIED implantations stratified by states, territories, and public and private sectors. **B:** Proportions of remote monitoring in CIEDs stratified by states, territories, and public and private sectors. CIED = cardiac implantable electronic device.

**Figure 3:**
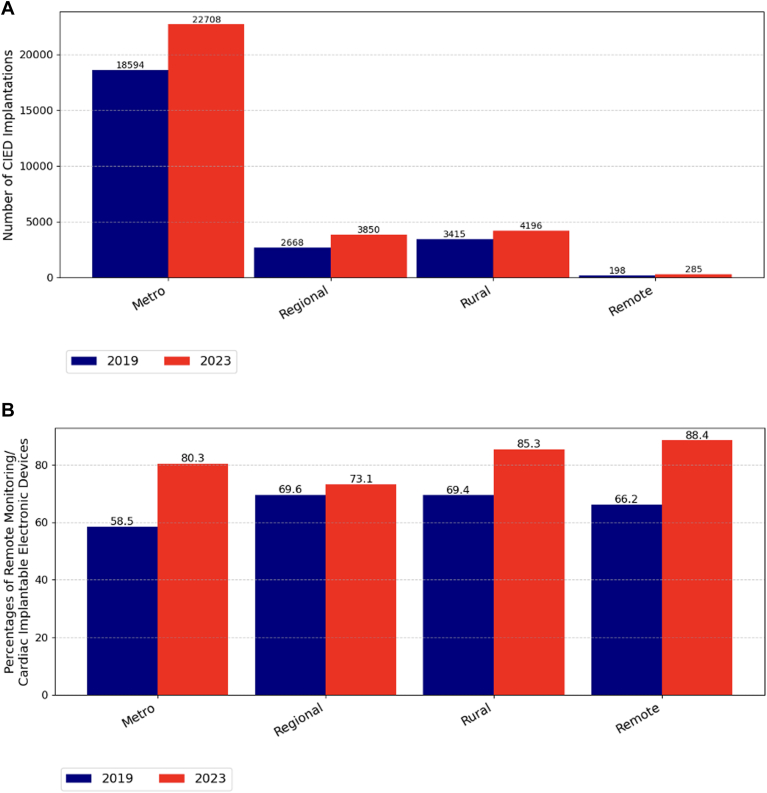
**A:** Number of CIED implants across geographic areas, in 2019 and 2023. **B:** RM allocation across geographic locations, in 2019 and 2023. CIED = cardiac implantable electronic device; RM = remote monitoring.

Data were analyzed for 32,145 CIED implants in 2019 and 37,437 in 2023. From 2019 to 2023, the total number of CIED implants in Australia increased by 16.5%, rising from 32,145 to 37,437 procedures (Figure 2A). Relative to population growth, this represents a 10.2% increase in the implantation rate between 2019 and 2023, rising from 125.9 to 138.8 implants per 100,000 people in Australia. Within the subset of data differentiated by insurance status, this increase seemed slightly steeper in the private sector (15,190–19,309; 27.1%) than the public sector (11,463–14,305; 24.8%). The greatest increases were seen for dual-chamber PPMs (15,641–20,959; 34.0%) and cardiac resynchronization therapies (CRTs) (3782–4381; 23.2%) (Supplemental Appendix 4). RM transmitter distribution increased from 75.5 to 117.6 RM transmitters distributed per 100,000 people, an increase of 55.8% relative to population growth, and this growth was consistent across all regions but persisted to be low use in the Northern Territory where the numbers are small (Figure 2B). For the subset of data that public and private sectors could differentiate, the largest increase in RM use was in the public sector, from 25.9% to 65.0%, but this was still lower than the private sector, which increased from 83.1% to 91.6% (Figure 2B). RM use was most common in remote and rural areas (88.4%) compared with urban areas (85.3%) (Figure 3B).

### CIED clinic data

A total of 50 CIED clinics in Australia and New Zealand completed the CIED clinic survey (Supplemental Appendix 5); 56% of Australian public clinics (31 of 55) responded to the distributed survey, 46% of New Zealand clinics (6 of 13) responded, and 13 Australian private clinics responded; however, it is unclear how many private clinics received the distributed survey (Supplemental Appendix 6). Clinics were surveyed from all states and territories in Australia, but there were no public clinic data for the Northern Territory and no private clinic data for South Australia and Western Australia. Nearly all CIED clinics employed cardiac physiologists (49 of 50 [98%]), many employing cardiologists (45 of 50 [90%]) and doctors-in-training (24 of 50 [48%]) and less frequently nurses (17 of 50 [34%]) and administrative staff (3/50 [6%]).

The median clinic patient load was greater in the public clinics (1402 patients) than the private clinics (1000 patients) in Australia; however, clinic patient loads in New Zealand were greater than in Australia (2920 patients) (Table 1). On average, CIED clinics reported that 30.2% of their workload was dedicated to managing RM (Table 1). Some CIED clinics have been operating RM services since 2008, with broader adoption by 2016. Most CIED clinics (39 of 42 [93%]) reported using RM for both scheduled reviews and alert-triggered reviews. Most clinics reported that RM alerts were monitored only on weekdays (20 of 21 [95% of Australian public clinics]), but approximately a third of Australian private and New Zealand clinics reported monitoring alerts on weekends, 31% (4 of 13) and 33% (2 of 6), respectively. Most clinics reported that cardiac physiologists were primarily responsible for monitoring RM alerts (90% [38 of 42]). Both CIED type and use of RM influenced the mean interval of time between scheduled in-person CIED follow-up visits, which was 10.0 months for patients with RM and 7.2 months for patients without RM (*P* ≤ .001). For CIED patients not using RM, implantable loop recorder (ILR), CRT, and implantable cardioverter-defibrillator (ICD) patients have approximately 6-month intervals between reviews, whereas PPM patients are reviewed on average every 10 months (Supplemental Appendix 7). PPM, ICD, and CRT patients who receive the RM service have a longer interval between in-person follow-up appointments than those who do not receive RM, 2.9, 3.8, and 3.0 months, respectively (all *P* < .001). There was no difference in in-person follow-up duration for ILR patients based on RM status (*P* = .89).

**Table 1:** CIED RM used by the CIED clinic in Australia and New Zealand

CIED RM management factor	Australia—public (n = 31)	Australia—private (n = 13)	New Zealand (n = 6)	Overall (n = 50)
Clinic patient load, median [IQR]	1402 [1903]	1000 [1252]	2920 [1677]	1303 [1820]
Clinics using the RM service	23/26 (88.5%)	13/13 (100%)	6/6 (100%)	42/45 (93.3%)
Year RM was commenced, median (IQR)	2016 (2012–2020)	2015 (2013–2019)	2010 (2010–2010)	2015 (2012–2019)
Proportion of CIED patients using RM, mean [SD]	58.7% [32.8%]	68.5% [25.3%]	40.5% [17.5%]	59.1% [29.7%]
How RM is used				
Scheduled CIED reviews	21/23 (91.3%)	13/13 (100%)	5/6 (83.3%)	39/42 (92.9%)
Alert-based reviews	21/23 (91.3%)	13/13 (100%)	6/6 (100%)	40/42 (95.2%)
Frequency of RM alert review				
Daily on weekdays and weekends	0/21 (0%)	4/13 (30.8%)	2/6 (33.7%)	6/40 (15%)
Daily on weekdays	20/21 (95.2%)	9/13 (69.2%)	4/6 (66.7%)	33/40 (82.5%)
Other: “When staff are available”	1/21 (4.8%)	0/13 (0%)	0/6 (0%)	1/40 (2.5%)
Staff types reviewing RM alerts				
Nurse	1/23 (4.3%)	1/13 (7.7%)	0/6 (0%)	2/42 (4.8%)
Physiologist	20/23 (86.9%)	12/13 (92.3%)	6/6 (100%)	38/42 (90.5%)
Doctor in training	0/23 (0%)	0/13 (0%)	0/6 (0%)	0/42 (0%)
Cardiologist	2/23 (8.7%)	3/13 (23.1%)	0/6 (0%)	5/42 (11.9%)
Third-party provider	1/23 (4.3%)	1/13 (7.7%)	0/6 (0%)	2/42 (4.8%)
CIED manufacturer representative	2/23 (8.7%)	3/13 (23.1%)	0/6 (0%)	5/42 (11.9%)
Proportion of clinic’s workload dedicated to RM, mean (SD)	33.0% (23.6)	27.7% (22.4)	25.9% (14.8)	30.2% (21.8)
Clinics providing RM service to rural and remote patients	21/29 (72.4%)	10/13 (76.9%)	6/6 (100%)	37/48 (77.1%)

CIED = cardiac implantable electronic device; IQR = interquartile range; RM = remote monitoring; SD = standard deviation.

## Discussion

Health services for patients with CIEDs are evolving rapidly. Alongside a steady rise in CIED implant rates, the proportion issued with RM has increased sharply. RM is workload intensive: clinics report dedicating approximately 30% of their time to RM, primarily managed by cardiac physiologists. In-person visits occur at significantly longer intervals for patients with ICDs, CRTs, and PPMs equipped with RM, suggesting that RM tasks may replace some face-to-face care.

### CIED/RM utilization

There was a steep incline in RM utilization in Australia between 2019 and 2023, consistent with international trends over the past decade.^9^^,^^10^ Although reduced in-person visits equate to health system benefits, these gains are offset by the substantial workload required to manage RM. This aligns with observations in Europe and the United States, where uptake is driven by patient and health care system benefits,^2^, ^3^, ^4^ reinforced by international recommendations,^11^, ^12^, ^13^ and accelerated by virtual care models during the coronavirus disease 2019 pandemic.^14^ RM use varies across Australia, likely reflecting differences in geography, funding, staffing, and interpretation of RM benefits. RM is widely used across all regions, with the highest uptake in rural and remote areas, which are typically underserved in cardiovascular care.^15^ This suggests that RM may improve access and equity, while underscoring the need for sustainable workflows in geographically diverse regions. RM use is increasing among publicly funded patients, but remains lower than private care, possibly owing to reduced willingness to pay, reimbursement gaps, and workload constraints in public clinics.^2^^,^^16^^,^^17^

### Workload

RM of CIEDs has mixed effects on clinic workload. A key benefit is reduced in-person review frequency, consistent with previous findings.^18^^,^^19^ This may improve convenience, lower travel costs, and enhance efficiency because scheduled visits rarely identify actionable issues.^20^ However, RM introduces substantial clinical and administrative tasks.^11^^,^^17^ Self-reported estimates from this study suggest that RM accounts for ∼30% of overall workload, aligning with international data,^21^ although this varies by clinic size, patient volume, staffing, and alert burden. Larger clinics or those with frequent unscheduled transmissions may dedicate more time to RM, whereas smaller clinics experience proportionally greater strain on staff resources. A European and North American workflow analysis reported that nonclinical RM tasks require 32 hours per week (17.5 minutes per patient, annually) with alert management as the main driver.^17^ CIED type influences workload: therapeutic devices require 0.6–1.2 hours per patient annually, whereas ILRs require 6.7–8.4 hours.^17^^,^^22^^,^^23^ Workload is likely to rise as patient complexity and device sophistication increase, necessitating improved tools, care models, and reimbursement. Future studies using time-tracking or workflow analysis could provide more objective estimates.

### Workforce

Cardiac physiologists are the primary staff type managing RM alerts in both public and private settings in Australia and New Zealand, consistent with UK and Italian models; in contrast, physician assistants or nurse practitioners fulfill this role in the United States.^9^^,^^16^ Unlike international practice, Australian cardiac physiologists are not mandated to register with the Australian Council for Clinical Physiologists.^24^ It is important to ensure that licensing and clinical professional development are in place to support and standardize the changing roles of cardiac physiologists in CIED RM.

Manufacturers provide post-CIED implant support, mainly in private clinics, and ad-hoc services in public clinics. Our survey data suggest a limited role in RM alert monitoring; however, this is contrary to data recently presented to the medical services advisory committee, in particular for private clinics.^5^ This discrepancy may be caused by a low survey response rate for private CIED clinics; thus, the authors reached out to the MTAA cardiac forum for further insight. Industry sources indicate that manufacturers support >55% of RM accounts and >40% of RM patients, with greater involvement in private clinics (>60%) than in public clinics (∼25%). Manufacturers monitor alerts year-round, underscoring their large contribution to RM delivery.

RM data are transmitted asynchronously, typically once daily, although frequencies vary by manufacturer. Guidelines offer limited direction on review timeliness; some recommend review only during business hours,^11^ as practiced by most clinics in this study. Some guidelines recommend out-of-hours monitoring,^25^ but this can create challenges related to staffing, liability, funding, and timely data access.^10^ Critically, there is no evidence that immediate review improves outcomes.

### Future models of care

The sharp rise in CIED implementation and RM provision underscores the need to review and adapt care models. Data collection challenges and observed heterogeneity suggest fragmented systems, with unclear accountability among hospital clinics, cardiologists, and manufacturers. Without reform, current models risk being overwhelmed. Future strategies should first include centralized, integrated, multidisciplinary RM-enabled virtual clinics to standardize follow-up and data management^26^; second, patient-centric systems leveraging digital health for engagement, education, and on-demand support^27^^,^^28^; and, third, registration frameworks for monitoring and accountability after implant.

### Limitations

There are some limitations to this study. Although all CIED manufacturers in Australia contributed, variability in data availability and formatting limited consistency and completeness. The lack of patient-level data prevented reporting findings disaggregated by age, sex, or comorbidity. Although 50 clinics participated, the total number of clinics supporting RM across Australia and New Zealand is unknown. Responses were predominantly from public clinics, providing limited insight into private-sector models, and the data collection method may not capture the contribution industry is making to RM data monitoring. Consequently, findings may not fully reflect the broader RM landscape.

### Implications and future needs

This study presents a multidimensional view of RM in Australia and New Zealand, highlighting rapid growth in CIED and RM use and associated workload implications. These trends demand evaluation of care models and future research on clinical outcomes, patient experience, service impact, and cost-effectiveness. Key priorities include (1) developing and testing workflow models to improve efficiency and outcomes, (2) accounting for geographic variation in RM provision when designing funding and service models, (3) conducting detailed workflow and cost analyses of RM reviews, (4) assessing the clinical benefits of continuous RM data, and (5) evaluating the feasibility of artificial intelligence driven alert management to reduce workload.

## Conclusion

This study provides the first in-depth analysis of CIED RM and associated health services in Australia and New Zealand, offering insights to guide future models of care. The rapid evolution of RM services also informs the design and implementation of other virtual care models using remotely monitored patient data.

## Data sharing

The deidentified data we analyzed are not publicly available, but requests to the corresponding author for the data will be considered on a case-by-case basis.

## Disclosures

The authors have no conflicts of interest to disclose.

## Footnotes

Appendix Supplementary data associated with this article can be found in the online version at https://doi.org/10.1016/j.hroo.2025.12.004.
