# Supplementary material for: Utilization and implementation of remote monitoring of cardiac implantable electronic devices in Australia and New Zealand: Adoption, workload, and integration challenges
Source: Heart Rhythm O2. 2025 Dec 13;7(2):335–43. doi: 10.1016/j.hroo.2025.12.004 (PMC12925928; doi:10.1016/j.hroo.2025.12.004)
Supplement: Supplementary appendix 1 [file mmc1.docx]

**CIED remote monitoring landscape analysis:**

**Industry data collection survey**

Version 1

Table of Contents

[Instructions 2](#_Toc158286114)

[Definitions 2](#_Toc158286115)

[Example output table - 2019 Incidence of RM from Private Hospital/Clinics 3](#_Toc158286116)

[Table 1. 2019 Incidence of RM from Private Hospital/Clinics 5](#_Toc158286117)

[Table 2. 2023 Incidence of RM from Private Hospital/Clinics 8](#_Toc158286118)

[Table 3. 2019 Incidence of RM from Public Hospital/Clinics 11](#_Toc158286119)

[Table 4. 2023 Incidence of RM from Public Hospital/Clinics 14](#_Toc158286120)

# **Instructions**

- Each CIED company will complete the four tables provided below
- The tables provided below are the ‘output’ tables to be completed by each CIED company, respectively. Upon completion the tables will be sent to an MTAA representative for ‘merging’.
- Definitions have been provided below on: (1) data collection timeframe, (2) patient postcode, (3) postcode grouping.
- If there are any questions on the data collection process or data definitions, please do not hesitate to contact the [REDACTED] team via [REDACTED]

# **Definitions**

- **Data collection timeframe:**
  - **Table 1:** 01/01/2019 – 31/12/2019
  - **Table 2:** 01/01/2023 – 31/12/2023
  - **Table 3:** 01/01/2019 – 31/12/2019
  - **Table 4:** 01/01/2023 – 31/12/2023
- **Patient postcode:** Postcode provided by patient at time of implant
- **Postcode grouping:**
  - Metropolitan postcodes as per attached excel file
  - Regional postcodes as per attached excel file
  - Rural postcodes as per attached excel file
  - Remote postcodes as per attached excel file

# **Example output table - 2019 Incidence of RM from Private Hospital/Clinics**

Below is an example of the outcome table, a written description has been provided to explain the data that is required in the each cell, e.g., column 2 row 3: please insert the total number of single chamber *PPM implanted in patients from metropolitan NSW/ACT during* 1/1/19 – 31/12/19

| **2019** (01/01/2019 – 31/12/2019) | **No. of single chamber PPM implants** | **No. of RM allocation for single chamber PPMs** | **No. of dual chamber PPM implants** | **No. of RM allocation for dual chamber PPMs** | **No. of ICD implants** | **No. of RM allocation for ICDs** | **No. of CRT implants** | **No. of RM allocation for CRT** | **No. of ILR implants** | **No. of RM allocation for ILR** |
| --- | --- | --- | --- | --- | --- | --- | --- | --- | --- | --- |
| **NSW/ACT** | | | | | | | | | | |
| Metro | *‘No. of* ***single*** *chamber PPM implanted in private patients from metropolitan NSW/ACT during 1/1/19 – 31/12/19 (based on patient postcode at time of implant)’* | *‘No. of RM transmitters allocated to private patients with a* ***single*** *chamber PPM in metropolitan NSW/ACT during 1/1/19 – 31/12/19*  *(based on patient postcode at time of implant)’* | *‘No. of* ***dual*** *chamber PPM implanted in private patients from metropolitan NSW/ACT during 1/1/19 – 31/12/19 (based on patient postcode at time of implant)’* | *‘No. of RM transmitters allocated to private patients with a* ***dual*** *chamber PPM in metropolitan NSW/ACT during 1/1/19 – 31/12/19*  *(based on patient postcode at time of implant)’* | *‘No. of* ***ICD*** *implanted in private patients from metropolitan NSW/ACT during 1/1/19 – 31/12/19 (based on patient postcode at time of implant)’* | *‘No. of RM transmitters allocated to private patients with an* ***ICD*** *in metropolitan NSW/ACT during 1/1/19 – 31/12/19*  *(based on patient postcode at time of implant)’* | *‘No. of* ***CRT*** *implanted in private patients from metropolitan NSW/ACT during 1/1/19 – 31/12/19 (based on patient postcode at time of implant)’* | *‘No. of RM transmitters allocated to private patients with a* ***CRT*** *in metropolitan NSW/ACT during 1/1/19 – 31/12/19*  *(based on patient postcode at time of implant)’* | *‘No. of* ***ILR*** *implanted in private patients from metropolitan NSW/ACT during 1/1/19 – 31/12/19 (based on patient postcode at time of implant)’* | *‘No. of RM transmitters allocated to private patients with a ILR in metropolitan NSW/ACT during 1/1/19 – 31/12/19*  *(based on patient postcode at time of implant)’* |
| Regional | Xx | xx | xx | xx | xx | xx | xx | xx | xx | xx |
| Rural | Xx | xx | xx | xx | xx | xx | xx | xx | xx | xx |
| Remote | Xx | xx | xx | xx | xx |  | xx | xx | xx | xx |

| **Table 1. 2019 Incidence of RM from Private Hospital/Clinics** | | | | | | | | | | |
| --- | --- | --- | --- | --- | --- | --- | --- | --- | --- | --- |
| **2019** (01/01/2019 – 31/12/2019) | **No. of single chamber PPM implants** | **No. of RM allocation for single chamber PPMs** | **No. of dual chamber PPM implants** | **No. of RM allocation for dual chamber PPMs** | **No. of ICD implants** | **No. of RM allocation for ICDs** | **No. of CRT implants** | **No. of RM allocation for CRT** | **No. of ILR implants** | **No. of RM allocation for ILR** |
| **NSW / ACT** | | | | | | | | | | |
| Metro | xx | xx | xx | xx | xx | xx | xx | xx | xx | xx |
| Regional | xx | xx | xx | xx | xx | xx | xx | xx | xx | xx |
| Rural | xx | xx | xx | xx | xx | xx | xx | xx | xx | xx |
| Remote | Xx | xx | xx | xx | xx | xx | xx | xx | xx | xx |
| **Vic** | | | | | | | | | | |
| Metro | xx | xx | xx | xx | xx | xx | xx | xx | xx | xx |
| Regional | xx | xx | xx | xx | xx | xx | xx | xx | xx | xx |
| Rural | xx | xx | xx | xx | xx | xx | xx | xx | xx | xx |
| Remote | xx | xx | xx | xx | xx | xx | xx | xx | xx | xx |
| **Qld** | | | | | | | | | | |
| Metro | xx | xx | xx | xx | xx | xx | xx | xx | xx | xx |
| Regional | xx | xx | xx | xx | xx | xx | xx | xx | xx | xx |
| Rural | xx | xx | xx | xx | xx | xx | xx | xx | xx | xx |
| Remote | xx | xx | xx | xx | xx | xx | xx | xx | xx | xx |
| **SA** | | | | | | | | | | |
| Metro | xx | xx | xx | xx | xx | xx | xx | xx | xx | xx |
| Regional | xx | xx | xx | xx | xx | xx | xx | xx | xx | xx |
| Rural | xx | xx | xx | xx | xx | xx | xx | xx | xx | xx |
| Remote | xx | xx | xx | xx | xx | xx | xx | xx | xx | xx |
| **WA** | | | | | | | | | | |
| Metro | xx | xx | xx | xx | xx | xx | xx | xx | xx | xx |
| Regional | xx | xx | xx | xx | xx | xx | xx | xx | xx | xx |
| Rural | xx | xx | xx | xx | xx | xx | xx | xx | xx | xx |
| Remote | xx | xx | xx | xx | xx | xx | xx | xx | xx | xx |
| **Tas** | | | | | | | | | | |
| Metro | xx | xx | xx | xx | xx | xx | xx | xx | xx | xx |
| Regional | xx | xx | xx | xx | xx | xx | xx | xx | xx | xx |
| Rural | xx | xx | xx | xx | xx | xx | xx | xx | xx | xx |
| Remote | xx | xx | xx | xx | xx | xx | xx | xx | xx | xx |
| **NT** | | | | | | | | | | |
| Metro | xx | xx | xx | xx | xx | xx | xx | xx | xx | xx |
| Regional | xx | xx | xx | xx | xx | xx | xx | xx | xx | xx |
| Rural | xx | xx | xx | xx | xx | xx | xx | xx | xx | xx |
| Remote | xx | xx | xx | xx | xx | xx | xx | xx | xx | xx |

# Table 2. 2023 Incidence of RM from Private Hospital/Clinics

| **2023** (01/01/2023 – 31/12/2023) | **No. of single chamber PPM implants** | **No. of RM allocation for single chamber PPMs** | **No. of dual chamber PPM implants** | **No. of RM allocation for dual chamber PPMs** | **No. of ICD implants** | **No. of RM allocation for ICDs** | **No. of CRT implants** | **No. of RM allocation for CRT** | **No. of ILR implants** | **No. of RM allocation for ILR** |
| --- | --- | --- | --- | --- | --- | --- | --- | --- | --- | --- |
| **NSW / ACT** | | | | | | | | | | |
| Metro | xx | xx | xx | xx | xx | xx | xx | xx | xx | xx |
| Regional | xx | xx | xx | xx | xx | xx | xx | xx | xx | xx |
| Rural | xx | xx | xx | xx | xx | xx | xx | xx | xx | xx |
| Remote | xx | xx | xx | xx | xx | xx | xx | xx | xx | xx |
| **Vic** | | | | | | | | | | |
| Metro | xx | xx | xx | xx | xx | xx | xx | xx | xx | xx |
| Regional | xx | xx | xx | xx | xx | xx | xx | xx | xx | xx |
| Rural | xx | xx | xx | xx | xx | xx | xx | xx | xx | xx |
| Remote | xx | xx | xx | xx | xx | xx | xx | xx | xx | xx |
| **Qld** | | | | | | | | | | |
| Metro | xx | xx | xx | xx | xx | xx | xx | xx | xx | xx |
| Regional | xx | xx | xx | xx | xx | xx | xx | xx | xx | xx |
| Rural | xx | xx | xx | xx | xx | xx | xx | xx | xx | xx |
| Remote | xx | xx | xx | xx | xx | xx | xx | xx | xx | xx |
| **SA** | | | | | | | | | | |
| Metro | xx | xx | xx | xx | xx | xx | xx | xx | xx | xx |
| Regional | xx | xx | xx | xx | xx | xx | xx | xx | xx | xx |
| Rural | xx | xx | xx | xx | xx | xx | xx | xx | xx | xx |
| Remote | xx | xx | xx | xx | xx | xx | xx | xx | xx | xx |
| **WA** | | | | | | | | | | |
| Metro | xx | xx | xx | xx | xx | xx | xx | xx | xx | xx |
| Regional | xx | xx | xx | xx | xx | xx | xx | xx | xx | xx |
| Rural | xx | xx | xx | xx | xx | xx | xx | xx | xx | xx |
| Remote | xx | xx | xx | xx | xx | xx | xx | xx | xx | xx |
| **Tas** | | | | | | | | | | |
| Metro | xx | xx | xx | xx | xx | xx | xx | xx | xx | xx |
| Regional | xx | xx | xx | xx | xx | xx | xx | xx | xx | xx |
| Rural | xx | xx | xx | xx | xx | xx | xx | xx | xx | xx |
| Remote | xx | xx | xx | xx | xx | xx | xx | xx | xx | xx |
| **NT** | | | | | | | | | | |
| Metro | xx | xx | xx | xx | xx | xx | xx | xx | xx | xx |
| Regional | xx | xx | xx | xx | xx | xx | xx | xx | xx | xx |
| Rural | xx | xx | xx | xx | xx | xx | xx | xx | xx | xx |
| Remote | xx | xx | xx | xx | xx | xx | xx | xx | xx | xx |

# Table 3. 2019 Incidence of RM from Public Hospital/Clinics

| **2019** (01/01/2019 – 31/12/2019) | **No. of single chamber PPM implants** | **No. of dual chamber PPM implants** | **No. of ICD implants** | **No. of CRT implants** | **No. of ILR implants** | **No. of RM transmitters purchased / allocated** |
| --- | --- | --- | --- | --- | --- | --- |
| **NSW/ACT** | | | | | | |
| Metro | xx | xx | xx | xx | xx | xx |
| Regional | xx | xx | xx | xx | xx | xx |
| Rural | xx | xx | xx | xx | xx | xx |
| Remote | xx | xx | xx | xx | xx | xx |
| **Vic** | | | | | | |
| Metro | xx | xx | xx | xx | xx | xx |
| Regional | xx | xx | xx | xx | xx | xx |
| Rural | xx | xx | xx | xx | xx | xx |
| Remote | xx | xx | xx | xx | xx | xx |
| **QLD** | | | | | | |
| Metro | xx | xx | xx | xx | xx | xx |
| Regional | xx | xx | xx | xx | xx | xx |
| Rural | xx | xx | xx | xx | xx | xx |
| Remote | xx | xx | xx | xx | xx | xx |
| **SA** | | | | | | |
| Metro | xx | xx | xx | xx | xx | xx |
| Regional | xx | xx | xx | xx | xx | xx |
| Rural | xx | xx | xx | xx | xx | xx |
| Remote | xx | xx | xx | xx | xx | xx |
| **WA** | | | | | | |
| Metro | xx | xx | xx | xx | xx | xx |
| Regional | xx | xx | xx | xx | xx | xx |
| Rural | xx | xx | xx | xx | xx | xx |
| Remote | xx | xx | xx | xx | xx | xx |
| **TAS** | | | | | | |
| Metro | xx | xx | xx | xx | xx | xx |
| Regional | xx | xx | xx | xx | xx | xx |
| Rural | xx | xx | xx | xx | xx | xx |
| Remote | xx | xx | xx | xx | xx | xx |
| **NT** | | | | | | |
| Metro | xx | xx | xx | xx | xx | xx |
| Regional | xx | xx | xx | xx | xx | xx |
| Rural | xx | xx | xx | xx | xx | xx |
| Remote | xx | xx | xx | xx | xx | xx |

# Table 4. 2023 Incidence of RM from Public Hospital/Clinics

| **2023** (01/01/2023 – 31/12/2023) | **No. of single chamber PPM implants** | **No. of dual chamber PPM implants** | **No. of ICD implants** | **No. of CRT implants** | **No. of ILR implants** | **No. of RM transmitters purchased / allocated** |
| --- | --- | --- | --- | --- | --- | --- |
| **NSW/ACT** | | | | | | |
| Metro | xx | xx | xx | xx | xx | xx |
| Regional | xx | xx | xx | xx | xx | xx |
| Rural | xx | xx | xx | xx | xx | xx |
| Remote | xx | xx | xx | xx | xx | xx |
| **Vic** | | | | | | |
| Metro | xx | xx | xx | xx | xx | xx |
| Regional | xx | xx | xx | xx | xx | xx |
| Rural | xx | xx | xx | xx | xx | xx |
| Remote | xx | xx | xx | xx | xx | xx |
| **QLD** | | | | | | |
| Metro | xx | xx | xx | xx | xx | xx |
| Regional | xx | xx | xx | xx | xx | xx |
| Rural | xx | xx | xx | xx | xx | xx |
| Remote | xx | xx | xx | xx | xx | xx |
| **SA** | | | | | | |
| Metro | xx | xx | xx | xx | xx | xx |
| Regional | xx | xx | xx | xx | xx | xx |
| Rural | xx | xx | xx | xx | xx | xx |
| Remote | xx | xx | xx | xx | xx | xx |
| **WA** | | | | | | |
| Metro | xx | xx | xx | xx | xx | xx |
| Regional | xx | xx | xx | xx | xx | xx |
| Rural | xx | xx | xx | xx | xx | xx |
| Remote | xx | xx | xx | xx | xx | xx |
| **TAS** | | | | | | |
| Metro | xx | xx | xx | xx | xx | xx |
| Regional | xx | xx | xx | xx | xx | xx |
| Rural | xx | xx | xx | xx | xx | xx |
| Remote | xx | xx | xx | xx | xx | xx |
| **NT** | | | | | | |
| Metro | xx | xx | xx | xx | xx | xx |
| Regional | xx | xx | xx | xx | xx | xx |
| Rural | xx | xx | xx | xx | xx | xx |
| Remote | xx | xx | xx | xx | xx | xx |
