# Supplementary material for: Utilization and implementation of remote monitoring of cardiac implantable electronic devices in Australia and New Zealand: Adoption, workload, and integration challenges
Source: Heart Rhythm O2. 2025 Dec 13;7(2):335–43. doi: 10.1016/j.hroo.2025.12.004 (PMC12925928; doi:10.1016/j.hroo.2025.12.004)
Supplement: Supplementary appendix 2 [file mmc2.docx]

**CIED remote monitoring landscape analysis:**

**CIED clinic data collection survey**

| **Details of person completing survey** |
| --- |

**Name:**

**Email:**

**Occupation:**

| 1. **Clinic details** |
| --- |

1. **Postcode location of the clinic:** _____________
2. **Where is the clinic based?**

*Please tick the category that best describes your service*

- 1. Public hospital
  2. Private rooms

Other: ­­­­­­­­­­_____________

1. **How many staff members work within the clinic?**
   1. Nurse _____
   2. Cardiac technician/Physiologist ______
   3. Doctor in training (Intern / Resident / Registrar / AT) _____
   4. Cardiologist _____
   5. Other _____
2. **How many CIED patients does the clinic currently service?________**
3. **Does the clinic service rural / remote patients?**
4. No
5. Yes
   1. If yes, what percentage of the clinic’s case load is this?
6. **CIED remote monitoring**
7. **Does the clinic use remote monitoring (RM) for ANY of their CIEDs?**
8. Yes
9. No
10. **Please estimate what year your clinic started using RM:** ___________
11. **How many clinic CIED patients currently receive RM? __________**
12. **Does the clinic monitor data (scheduled reviews and/or alerts) for:**

*Please tick the all relevant categories*

- 1. Privately implanted patients
  2. Publicly implanted patients with private health fund cover
  3. Publicly implanted patients with medicare cover

1. **Are all CIED patients offered the RM service?**
   1. **Yes**
   2. **No**
2. **If ‘No’ to Q10 - Does the clinic have criteria to determine which patients are offered RM (e.g., CIED type, geographical location, insurance coverage, nursing home patients, other etc)?**

*Please tick the category that best describes your service*

1. No
2. Yes

If Yes, please outline the criteria:________________

1. **How is RM used in the clinic?**

*Please tick all relevant categories*

1. Scheduled routine CIED reviews (e.g., every 6 or 12 months)
2. Alert-based reviews (unscheduled transmissions)

Other: ___________

1. **How many clinic staff are trained/able to manage RM alerts (Unscheduled transmissions)?**

*Please complete for all relevant staff*

- 1. Nurse _____
  2. Cardiac technician ______
  3. Non-specialist doctor (Intern / Resident / Registrar / AT) _____
  4. Cardiologist _____
  5. Other _____
  6. Does not employ own staff

1. **If the clinic monitors for alerts; who is initially checking these RM alerts?**

*Please tick all relevant categories*

1. Nurse
2. Cardiac technician / physiologist
3. Doctor in training (Intern / Resident / Registrar / AT)
4. Cardiologist
5. Third party provider (Independent technical Service Provider)
6. Device manufacturer representative
7. Other:______________
8. **Currently how frequently does the clinic review RM alerts per week?**

*Please tick the category that best describes your service*

1. Daily on weekdays
2. Less than daily on weekdays
3. Daily on weekdays and weekends
4. Other: ___________
5. **Are RM alerts monitored outside business hours?**

*Please tick the category that best describes your service*

- 1. No
  2. Yes
     1. If yes, are the monitored by:

*Please tick all relevant categories*

- - 1. Nurse
    2. Cardiac technician / physiologist
    3. Doctor in training (Intern / Resident / Registrar / AT)
    4. Cardiologist
    5. Third party provider (Independent technical Service Provider)
    6. Device manufacturer representative

1. **On average, how much staff FTE are put towards managing CIED RM for each staff member? (E.g. Set-up, patient education, alert-monitoring, alert management, scheduled reviews)**

*Please complete for all relevant staff*

- 1. Nurse _____
  2. Cardiac technician / physiologist ______
  3. Doctor in training (Intern / Resident / Registrar / AT) _____
  4. Cardiologist _____
  5. Other _____
  6. Does not employ own staff

1. **Please complete the below table on frequency of SCHEDULED review type for each CIED type**

| **Review type** | **Frequency** |
| --- | --- |
| **PPM patients with RM** | |
| Scheduled routine RM reviews performed | *E.g., 6 monthly* |
| Scheduled in-person reviews performed |  |
| **PPM patients WITHOUT RM** | |
| Scheduled in-person reviews performed |  |
| **ICD patients with RM** | |
| Scheduled routine RM reviews performed |  |
| Scheduled in-person reviews performed |  |
| **ICD patients WITHOUT RM** | |
| Scheduled in-person reviews performed |  |
| **CRT patients with RM** | |
| Scheduled routine RM reviews performed |  |
| Scheduled in-person reviews performed |  |
| **CRT patients WITHOUT RM** | |
| Scheduled in-person reviews performed |  |
| **ILR patients with RM** | |
| Scheduled routine RM reviews performed |  |
| Scheduled in-person reviews performed |  |
| **ILR patients WITHOUT RM** | |
| Scheduled in-person reviews performed |  |

| 1. **CIED clinic funding** |
| --- |

1. How frequent does the clinic claim the available MBS codes for CIED and RM reviews, listed below?

*Please tick the category that best describes your service for each row*

| **MBS code** | **Never** | **Occasionally** | **Sometimes** | **Frequently** | **Always** |
| --- | --- | --- | --- | --- | --- |
| Remote CIED review of alerts **(Item numbers: 11719, 11725** |  |  |  |  |  |
| In-person clinic CIED checks following abnormality detection **(Item numbers: 11720, 11726)** |  |  |  |  |  |
| In-person clinic CIED checks for scheduled reviews **(Item numbers: 11721, 11727** |  |  |  |  |  |
| in ILR checks for the investigation of atrial fibrillation for those implanted to investigate the cause of an embolic stroke (**Item numbers: 11728, 11736)** |  |  |  |  |  |
| in ILR checks for the investigation of cryptogenic stroke or recurrent unexplained syncope **(Item numbers: 11731, 11737)** |  |  |  |  |  |

1. **Are patients charged an out-of-pocket fee when attending the clinic?**

*Please tick the category that best describes your service*

1. Yes
2. No
3. **What percentage of your overall Remote Monitoring service costs (Alert reviews, scheduled transmissions, clinic infrastructure, staff, equipment) are covered by the following reimbursement streams?**

*Please tick the category that best describes your service*If **PUBLIC** Hospital Clinic (percentages should add up to 100%)

Absorbed in department budget:                                     __%

MBS Item code fees for RM of CIEDs                              __%

Dedicated RM budget                                                         __%

Other, please describe:     _______________               __%

If **PRIVATE** Clinic *(percentages should add up to 100%)*

Absorbed in practice general expenses                          __%

MBS Item code fees for RM of CIEDs                              __%

Patient out of pocket fees                                                 __%

Other, please describe:     ______________                  __%

| 1. **Electronic database** |
| --- |

1. **Do you maintain an electronic database of clinic patients that allows you to characterise your RM clinic?**
   1. Yes:

The database covers: *Please tick all relevant categories*

- - - Patient demographics
    - CV RF
    - Number of patients offered RM
    - Number of patients utilising RM
    - RM alerts received
    - Actions post receiving alerts
      - Investigations ordered
      - In-person review
      - Prompting ED presentation
    - Results of investigations ordered
    - Time between receiving alerts and initial action
    - Other: _____
  1. No

1. If yes, did you use the electronic database to answer this questionnaire?
   1. Yes
   2. No
2. If yes, what is the electronic database is used for:

*Please tick all relevant categories*

- 1. Audit purposes
  2. Research purposes
  3. Patient management
