# Supplementary material for: Utilization and implementation of remote monitoring of cardiac implantable electronic devices in Australia and New Zealand: Adoption, workload, and integration challenges
Source: Heart Rhythm O2. 2025 Dec 13;7(2):335–43. doi: 10.1016/j.hroo.2025.12.004 (PMC12925928; doi:10.1016/j.hroo.2025.12.004)
Supplement: Supplementary appendix 3 [file mmc3.pdf]

THE UNIVERSITY OF SYDNEY

Research Integrity & Ethics Administration
Human Research Ethics Committee

Tuesday, 21 November 2023

[REDACTED]

Dear [REDACTED],

Project Title: CIED remote monitoring Australian landscape analysis

The NHMRC National Statement on Ethical Conduct in Human Research ("The National Statement") outlines circumstances where research that carries only negligible risk may be exempted from ethical review.

The National Statement defines negligible risk: *"The expression 'negligible risk research' describes research in which there is no foreseeable risk of harm or discomfort; and any foreseeable risk is no more than inconvenience." (National Statement 2.1.7)*

Further, the National Statement states that institutions may choose to exempt research from ethical review which meets the following criteria:

(a) is negligible risk research (as defined in paragraph 2.1.7); and
(b) involves the use of existing collections of data or records that contain only non-identifiable data about human beings." (*National Statement 5.1.22*)

Based on what you have described in email communications to the Ethics Office, your project is considered negligible risk as it meets both of the above criteria.

Should any future work not comply with any of the above criteria, the project must be submitted for ethical review prior to commencing research. Please note that retrospective ethical approval of research cannot be given by the HREC.

Please contact the Ethics Office should you require further information or clarification.

Sincerely,

Kate Lowrie
Human Ethics Officer

Research Integrity & Ethics Administration
Research Portfolio
F23 Michael Spence Building
The University of Sydney
NSW 2006 Australia

T +61 2 90369161
E human.ethics@sydney.edu.au
W sydney.edu.au/ethics

ABN 15 211 513 464
CRICOS 00026A
