# Supplementary material for: Utilization and implementation of remote monitoring of cardiac implantable electronic devices in Australia and New Zealand: Adoption, workload, and integration challenges
Source: Heart Rhythm O2. 2025 Dec 13;7(2):335–43. doi: 10.1016/j.hroo.2025.12.004 (PMC12925928; doi:10.1016/j.hroo.2025.12.004)
Supplement: Supplementary appendix 4 [file mmc4.docx]

|  | **Single Chamber PPM** | | **Dual Chamber**  **PPM** | | **ICD** | | **CRT** | | **ILR** | | **Total** | |  |
| --- | --- | --- | --- | --- | --- | --- | --- | --- | --- | --- | --- | --- | --- |
|  | **2019** | **2023** | **2019** | **2023** | **2019** | **2023** | **2019** | **2023** | **2019** | **2023** | **2019** | **2023** |  |
| **No. of implants** | 4005 | 4204 | 15641 | 20959 | 3782 | 3863 | 3554 | 4381 | 5163 | 6759 | 32145 | 37437 |  |
|  | | | | | | | | | | | | | |
