# Supplementary material for: Utilization and implementation of remote monitoring of cardiac implantable electronic devices in Australia and New Zealand: Adoption, workload, and integration challenges
Source: Heart Rhythm O2. 2025 Dec 13;7(2):335–43. doi: 10.1016/j.hroo.2025.12.004 (PMC12925928; doi:10.1016/j.hroo.2025.12.004)
Supplement: Supplementary appendix 5 [file mmc5.docx]

**Supplementary Appendix 5.** Details of clinics that completed the survey

|  | **Australia Public (n=31)** | **Australia Private (n=13)** | **New Zealand**  **(n=6)** | **Overall**  **(n=50)** |
| --- | --- | --- | --- | --- |
| **Location** | | | | |
| NSW/ACT | 9/31 (29%) | 6/13 (46.2%) | NA | 15/50 (30%) |
| QLD | 9/31 (29%) | 3/13 (23.1%) | NA | 12/50 (24%) |
| Vic | 8/31 (25.8%) | 1/13 ((7.7%) | NA | 9/50 (18%) |
| SA | 2/31 (6.5%) | 0/13 (0%) | NA | 2/50 (4%) |
| WA | 2/31 (6.5%) | 0/13 (0%) | NA | 2/50 (4%) |
| Tas | 1/31 (3.2%) | 1/13 (7.7%) | NA | 2/50 (4%) |
| NT | 0/31 (0%) | 2/13 (15.4%) | NA | 2/50 (4%) |
| New Zealand | NA | NA | 6/6 (100%) | 6/50 (12%) |
| **Staff types employed in clinic** | | | | |
| Nurses | 9/31 (29%) | 7/13 (53.8%) | 1/6 (16.7%) | 17/50 (34%) |
| Physiologists | 30/31 (96.8%) | 13/13 (100%) | 6/6 (100%) | 49/50 (98%) |
| Doctors in training | 20/31 (64.5%) | 3/13 (23.1%) | 3/6 (50%) | 24/50 (48%) |
| Cardiologists | 29/31 (93.5%) | 12/13 (92.3%) | 4/6 (66.7%) | 45/50 (90%) |
| Administrative | 2/31 (6.5%) | 1/13 (7.7%) | 0/6 (0%) | 3/50 (6%) |
| **Clinic patient load (mean [SD])** | 1672 [1327] | 1563 [1471] | 3048 [1630] | 1817 [1454] |
| NA: Not applicable | | | | |
