# Supplementary material for: Utilization and implementation of remote monitoring of cardiac implantable electronic devices in Australia and New Zealand: Adoption, workload, and integration challenges
Source: Heart Rhythm O2. 2025 Dec 13;7(2):335–43. doi: 10.1016/j.hroo.2025.12.004 (PMC12925928; doi:10.1016/j.hroo.2025.12.004)
Supplement: Supplementary appendix 6 [file mmc6.docx]

**Supplementary appendix 6:** Survey response rates for Australian public clinics, stratified by state/territories, Australian private clinics and New Zealand clinics.

| **CIED Clinics** | **Survey Responders**  **n/N (%)** |
| --- | --- |
| **Public Clinics** | |
| NSW/ACT | 9/21 (43%) |
| QLD | 9/10 (90%) |
| Vic | 8/13 (62%) |
| SA | 2/3 (67%) |
| WA | 2/5 (40%) |
| Tas | 1/2 (3.2%) |
| NT | 0/1(0%) |
| Overall | 31/55 (56%) |
| **Private Clinics** | 13/NA* |
| **New Zealand** | 6/13(46.1%) |
| n = Number of responders  N= Number of clinics the survey was distributed to  * Denominator is unknown as the survey was disseminated by organisations – rather than individual contact. Most private cardiologists in Australia are sole traders and hence it is It is also difficult to identify which private clinics have a dedicated CIED clinic | |
