# Supplementary material for: Utilization and implementation of remote monitoring of cardiac implantable electronic devices in Australia and New Zealand: Adoption, workload, and integration challenges
Source: Heart Rhythm O2. 2025 Dec 13;7(2):335–43. doi: 10.1016/j.hroo.2025.12.004 (PMC12925928; doi:10.1016/j.hroo.2025.12.004)
Supplement: Supplementary appendix 7 [file mmc7.docx]

**Supplementary appendix 7.** Interval (months) between scheduled in-person CIED follow-up visits based on CIED type and if they have RM

|  | **Australia – Public n=31** | **Australia – Private n=13** | **New Zealand n=6** | **Overall**  **n=50** |
| --- | --- | --- | --- | --- |
| **All CIEDs** |  |  |  |  |
| RM, Mean (SD) ^1^ | 10.7 (5.3) | 9.3 (2.9) | 9.1 (2.0) | 10.0 (4.3) |
| Without RM, Mean (SD) ^1^ | 7.4 (1.6) | 7.0 (2.0) | 6.3 (2.4) | 7.2 (1.8) |
| **PPM** | | | | |
| RM, Mean (SD)^1^ | 13.0 (6.7) | 11.1 (3.8) | 13 (5.9) | 12.4 (5.7) |
| Without RM, Mean (SD) ^1^ | 10.1 (2.3) | 8.5 (2.5) | 9.0 (3.3) | 9.5 (2.5) |
| **ICD** | | | | |
| RM, Mean (SD) ^1^ | 10.2 (6.9) | 8.8 (3.1) | 12.0 (0) | 10.0 (5.3) |
| Without RM, Mean (SD) ^1^ | 5.8 (0.5) | 6.8 (1.9) | 6.5 (2.9) | 6.2 (1.5) |
| **CRT** | | | | |
| RM, Mean (SD) ^1^ | 9.5 (6.2) | 8.8 (3.1) | 9.5 (2.9) | 9.2 (4.9) |
| Without RM, Mean (SD) ^1^ | 6.4 (1.3) | 6.8 (1.9) | 4.5 (1.6) | 6.2 (1.7) |
| **ILR** | | | | |
| RM, Mean (SD) ^1^ | 6.3 (5.2) | 7.6 (3.9) | 0.0 (0) | 5.7 (4.9) |
| Without RM, Mean (SD) ^1^ | 6.4 (3.5) | 5.5 (2.6) | 4.8 (4.5) | 5.9 (3.4) |
| ^1^Months | | | | |
